# Increased cysteine metabolism in PINK1 models of Parkinson's disease

**DOI:** 10.1242/dmm.049727

**Published:** 2023-01-25

**Authors:** Marco Travaglio, Filippos Michopoulos, Yizhou Yu, Rebeka Popovic, Edmund Foster, Muireann Coen, L. Miguel Martins

**Affiliations:** ^1^ MRC Toxicology Unit, University of Cambridge, Gleeson Building, Tennis Court Road, Cambridge CB2 1QR, UK; ^2^ Oncology Safety, Clinical Pharmacology & Safety Sciences, R&D, AstraZeneca, Cambridge, UK; ^3^ Oncology R&D, Research & Early Development, AstraZeneca, Cambridge, UK; ^4^ Neuroscience Safety, Clinical Pharmacology & Safety Sciences, R&D, AstraZeneca, Cambridge, UK; ^5^ Department of Metabolism, Digestion and Reproduction, Faculty of Medicine, Imperial College, London, UK

**Keywords:** *Drosophila*, Stem cell research, PINK1, Mitochondria, Metabolism, Parkinson's disease

## Abstract

Parkinson's disease (PD), an age-dependent neurodegenerative disease, is characterised by the selective loss of dopaminergic neurons in the substantia nigra (SN). Mitochondrial dysfunction is a hallmark of PD, and mutations in *PINK1*, a gene necessary for mitochondrial fitness, cause PD. *Drosophila melanogaster* flies with *pink1* mutations exhibit mitochondrial defects and dopaminergic cell loss and are used as a PD model. To gain an integrated view of the cellular changes caused by defects in the PINK1 pathway of mitochondrial quality control, we combined metabolomics and transcriptomics analysis in *pink1*-mutant flies with human induced pluripotent stem cell (iPSC)-derived neural precursor cells (NPCs) with a *PINK1* mutation. We observed alterations in cysteine metabolism in both the fly and human PD models. Mitochondrial dysfunction in the NPCs resulted in changes in several metabolites that are linked to cysteine synthesis and increased glutathione levels. We conclude that alterations in cysteine metabolism may compensate for increased oxidative stress in PD, revealing a unifying mechanism of early-stage PD pathology that may be targeted for drug development.

This article has an associated First Person interview with the first author of the paper.

## Teaser

**Summary:** Increased cysteine metabolism in Parkinson's disease models.

## INTRODUCTION

Parkinson's disease (PD) is the second most common neurodegenerative disorder, affecting 1% of the population over 60 years old worldwide (Tysnes and Storstein, 2017). Clinically, PD is characterised by the progressive appearance of motor disturbances, which are largely attributed to the selective death of dopaminergic neurons in the substantia nigra pars compacta (SNpc). To date, research into the aetiology of PD has relied upon the use of toxin or transgenic animal models to explore key aspects of disease development and progression. The value of these models rests on the premise that the selective death of dopaminergic neurons is linked to molecular cascades that are triggered by a range of substances and events, including neurotoxins or the manipulation of disease-related genes in animal models (Bezard et al., 2013). However, the high failure rate of recent clinical trials has highlighted major limitations of current animal studies, questioning the validity of these approaches to address the complexity of PD (Outeiro et al., 2021). Given the elusive knowledge of factors that concur with the onset and progression of the disease and the high attrition rate, new approaches to identify the molecular fingerprints linked to PD diagnosis and progression are urgently needed.

Mitochondrial dysfunction is a hallmark of PD, and defects in the cellular mechanisms that ensure mitochondrial health play a key role in disease onset and development (reviewed by Celardo et al., 2014). Mutations in a gene encoding the mitochondrial PTEN-induced kinase 1 (PINK1) lead to the accumulation of defective mitochondria and cause a form of familial PD (Valente et al., 2004). Individuals carrying *PINK1* mutations are characterised by an early-onset and slowly progressing form of PD, and most of their symptoms are often indistinguishable from patients with idiopathic PD (Schneider and Klein, 2018). On average, PD patients with recessive mutations in *PINK1* also exhibit a better response to l-3,4-dihydroxyphenylalanine (L-DOPA) than idiopathic Parkinson's disease (IPD) patients, although drug-related dyskinesias and fluctuations of symptoms often occur earlier than IPD (Valente and Ferraris, 2010). Post-mortem analyses of brains from PD patients indicate the presence of neuronal inclusions called Lewy bodies (reviewed by Gibb and Lees, 1988). However, it is unclear to what degree patients with monogenic forms of PD – caused by mutations in *PINK1* or E3 ubiquitin protein ligase (*PRKN*, hereafter referred to as *Parkin*), two components of a mitochondrial quality-control mechanism – have these neuronal inclusions.

The fruit fly *Drosophila melanogaster* is a powerful model of PD. *Drosophila pink1*-mutants show mitochondrial defects and an age-dependent degeneration of dopaminergic neurons (Park et al., 2006). Mitochondrial dysfunction in *pink1*-mutant flies causes global metabolic changes (Tufi et al., 2014), including alterations in several pathways involved in amino acid metabolism (Celardo et al., 2017).

Recently, the development of induced pluripotent stem cell (iPSC) technologies has provided a unique opportunity to investigate disease mechanisms linked to *PINK1* mutations in a genetically robust system. Although these technologies remain hampered by phenotypic variability and batch-to-batch variations, a recently developed method for genome editing using clustered regularly interspaced short palindromic repeats (CRISPR)–Cas9 provides a means to generate clonal cell lines that are isogenic, thereby overcoming some shortcomings of conventional iPSC approaches. The availability of genetically defined pairs of disease and control cell lines has been crucial in distinguishing PD-related phenotypes from genetic background noise (Novak et al., 2022; Reinhardt et al., 2013).

Consistent with the clinical presentation of homozygous *PINK1* loss-of-function, the I368N mutation of *PINK1* causes early-onset and slowly progressive PD that starts at a relatively young age, i.e. at ∼30 years (Ando et al., 2017). Functional characterisation of iPSC-derived cells that carry this mutation has highlighted a selective alteration in mitophagy due to reduced interaction between PINK1 and the chaperone Hsp90 (Ando et al., 2017). However, a systematic characterisation of the mitochondrial defects linked to this mutation has not been completed to date.

Here, we combined exploratory approaches involving metabolomics and transcriptomics in two models of PD that are associated with mutations in *PINK1*. Using both *Drosophila* and iPSC-derived human neural precursor cells (NPCs) carrying a *PINK1* I368N mutation, we found that mitochondrial defects associated with the loss in PINK1 activity cause alterations in sulphur metabolism.

We found that cysteine, a sulphur-containing amino acid, is the most downregulated amino acid in *pink1*-mutant flies, indicating that cysteine degradation is increased in this PD model. This finding was consistent with global metabolic changes in *PINK1* I368N NPCs, in which a Warburg-type metabolic shift coincided with increased levels of glutathione and alterations in cysteine metabolites. Cysteine is widely used throughout the cell for several reactions that are involved in catalysis, trafficking and response to oxidative stress. This amino acid contains a sulphur-comprising moiety that acts as a nucleophilic thiol (-SH) group and is readily oxidised. Under physiological conditions, protein synthesis accounts for most of the cysteine usage; however, under conditions of high oxidative stress, cysteine is necessary for the synthesis of glutathione (Courtney-Martin and Pencharz, 2016), the most important antioxidant in cells.

Our results, for which data obtained from a fruit fly model of PD and PD patient primary cells were combined, showed a convergence of disease-related phenotypes between the two models of PD. We propose that alterations in cysteine metabolism are part of a metabolic response that is triggered by mutations in *PINK1* to counteract the high levels of reactive oxygen species (ROS) that are generated by defective mitochondria. Our data support the feasibility of a network-based, multiomics approach to generate disease-related hypotheses that can later be tested in primary cells or animal models of human diseases. Our analysis points to a common disease mechanism that could be exploited for therapeutic intervention.

## RESULTS

### Combined metabolic and transcriptomics analysis of *pink1*-mutant flies reveals alterations in cysteine metabolism

**Fig. 1.**
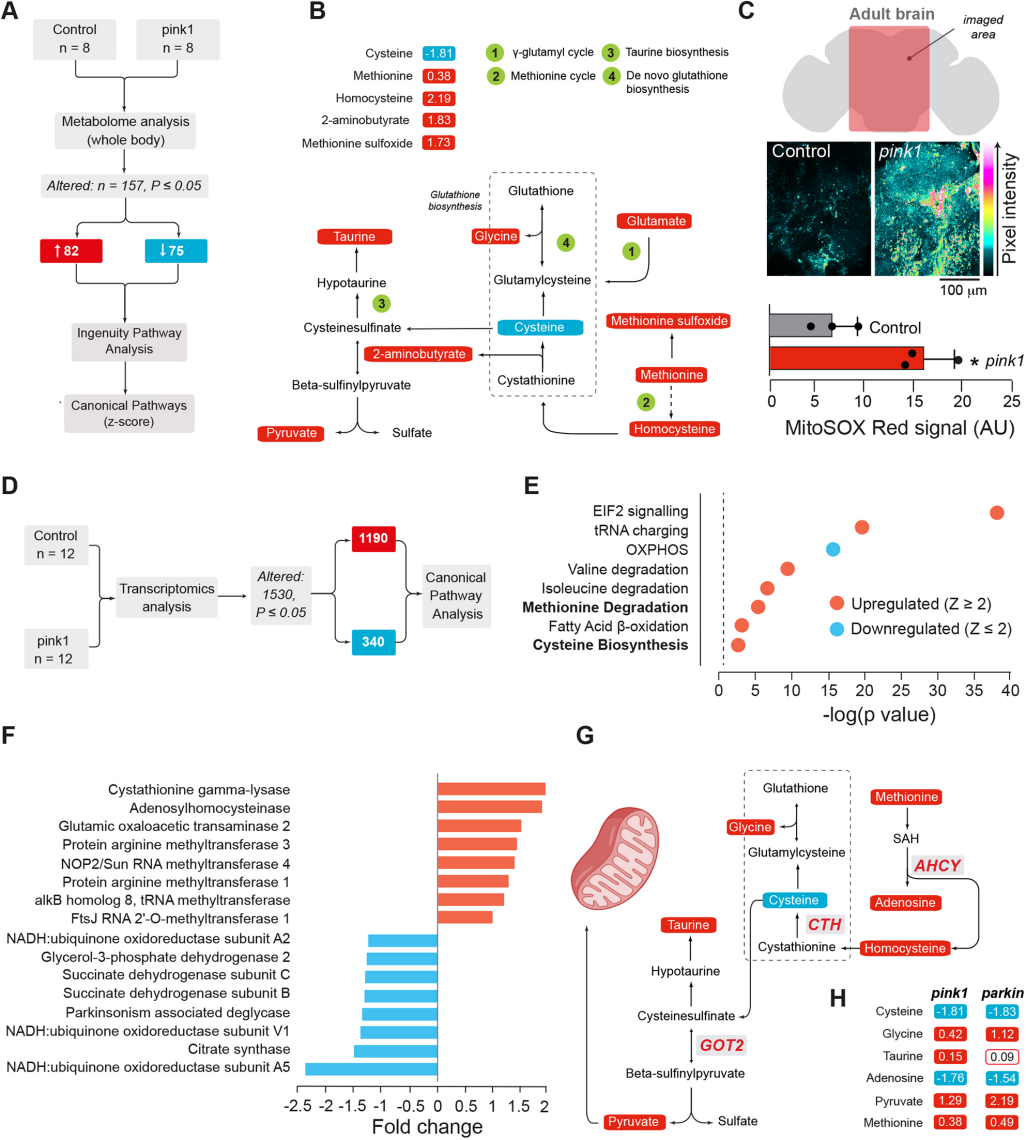
***Pink1*-mutant flies show alterations in cysteine metabolism.** (A) Workflow used to identify metabolic alterations in *Drosophila pink1*-mutant flies. The canonical pathways algorithm (Ingenuity Pathway Analysis) was used to determine changes in amino acid metabolism pathways. (B) Coordinated changes in the network of cysteine metabolites detected in *pink1*-mutant flies. Red or blue correspond to the metabolites that were significantly up- or downregulated, respectively (*P*≤0.05). The statistical significance was determined using Welch's two-sample *t*-test (*n*=8). Numbers encircled in green denote relevant pathways linked to cysteine metabolism. (C) Increased mitochondrial reactive oxygen species (ROS) in the brain of *pink1*-mutant flies. Top: Schematic of analysed brain area. Middle: Representative confocal images. Bottom: Quantitative analysis of mitochondrial ROS by using a MitoSOX Red mitochondrial superoxide indicator in control and *pink1* genotypes. Intensity levels are visualised using a five-tone heatmap. Data are shown as the mean±s.e.m. (*n*=3 per genotype; asterisks, two-tailed Student's *t*-test, *P<0.05). (D) Workflow used to identify transcriptome changes in *pink1-*mutant flies. (E) Analysis of the canonical pathways in *Drosophila pink1*-mutant flies. Red and blue circles correspond to pathways (as shown at the *y*-axis) that are significantly up- (Z≥2) or downregulated (Z≤−2). (F) Plotted is the upregulation of pathway components for cysteine biosynthesis (red) and the concomitant downregulation of components relevant in the mitochondrial electron transport chain (blue) in *pink1*-mutant flies. (G) Schematic of the integrated metabolomics and transcriptomics alterations in the cysteine metabolism in *Drosophila pink1* mutants. Three transcripts involved in synthesis and degradation of cysteine, i.e. cystathionine gamma-lyase (CTH), adenosyl homocysteinase (AHCY) and aspartate aminotransferase 2 (GOT2)] are upregulated in *pink1*-mutant flies (log2-fold change ≥1.5). (H) Comparison of metabolic alterations in the cysteine metabolism of *pink1*-mutant or *parkin*-mutant flies. The red outline corresponds to a comparison with lower statistical significance (0.05<*P*<0.10). The statistical significance was determined using Welch's two-sample *t-test* (*n*=8). Genotypes used: *w; +; daGAL4/+* (control) and *pink1^B9^; +; +^,^* (pink1) for A-G; *w1118*; +; +(control) and *w; +; park^25^* (parkin) for H. Metabolites significantly upregulated are indicated in red; metabolites significantly downregulated blue; *P*≤0.05 (A,B,D-H).

*Drosophila pink1* mutants accumulate defective mitochondria due to defects in mitophagy, a pathway used to recycle defective mitochondria. The mitochondrial defects in these mutants reprogram the metabolome (Celardo et al., 2017; Tufi et al., 2014), shut down translation (Celardo et al., 2016) and cause an accumulation of free amino acids (Celardo et al., 2017), the building blocks for proteins. Notably, cysteine, an amino acid with a highly reactive thiol group, does not accumulate in *Drosophila pink1* mutants but, rather, dissipates(Celardo et al., 2017). To reveal why cysteine does not follow the trend of global amino acid alterations in *pink1*-mutant flies, we first conducted a bioinformatics analysis by using Ingenuity Pathway Analysis (IPA) (Fig. 1A). The *canonical pathway* algorithm in IPA confirmed that cysteine was the most downregulated amino acid in *pink1*-mutant flies (Fig. 1B). We also observed an increase in the sulphur-containing metabolites methionine and homocysteine, and detected higher levels of 2-aminobutyrate, a downstream component of cysteine metabolism (Fig. 1B). By further exploring global metabolic changes in cysteine metabolism, we detected a significant increase in several by-products of cysteine degradation, including taurine and pyruvate (Fig. 1B). The observed accumulation of biochemicals associated with cysteine biosynthesis is consistent with an increased utilisation of cysteine by *pink1*-mutant flies. Cysteine is the rate-limiting substrate in the synthesis of glutathione, a powerful antioxidant. Although we found neither reduced nor oxidised glutathione in our analysis, several biochemicals associated with glutathione recycling, including gamma-glutamyl-leucine and the amino acid derivate 5-oxoproline (also known as pyroglutamic acid) had been detected previously (Tufi et al., 2014). Next, to test whether the changes in cysteine metabolism could reflect an increased oxidative environment, we measured mitochondrial ROS levels in *pink1*-mutant flies and found a significant increase in their levels (Fig. 1C). These observations indicate that cysteine depletion in *pink1*-mutant flies is due to an increased need for glutathione biosynthesis to mitigate increased oxidative stress.

Next, we determined whether the alterations in cysteine metabolites are coupled with an altered transcriptional signature for cysteine metabolism enzymes in *pink1*-mutant flies. Based on an analysis of transcript levels encoding enzymes in cysteine metabolism (see Fig. 1D for the analysis workflow), we detected alterations in several pathways, including those of methionine degradation and cysteine biosynthesis (Fig. 1E). Transcripts that encoded pathway components for methionine degradation and cysteine biosynthesis were among those most upregulated in *pink1*-mutant flies. By contrast, transcripts encoding pathway components needed for the mitochondrial oxidative phosphorylation (OXPHOS) system were downregulated (Fig. 1F). Individual pathway analysis also showed an increase in several proteins involved in cysteine metabolism, including cystathionine gamma-lyase (CTH) and adenosylhomocysteinase (AHCY) and aspartate aminotransferase (GOT2) (Fig. 1G). Because *PINK1* operates upstream of the PD-linked ubiquitin E3 ligase Parkin (Park et al., 2006) we next looked at components of the cysteine metabolism network in *parkin* (officially known as *park*) mutant flies. Global metabolic profiling of these mutants revealed broad alignment of metabolic alterations between *pink1* and *parkin* mutant flies (Fig. 1H), suggesting that alterations in cysteine metabolism are a common feature linked to the disruption of the PINK1/Parkin pathway of mitochondrial quality control.

In summary, this combined approach revealed a selective upregulation of the transcriptional and metabolomics networks involved in cysteine biosynthesis in *pink1*-mutant flies, extending the range of metabolic alterations observed (Tufi et al., 2014) in this fly model of PD.

### Generation of NPCs from a PD patient carrying a *PINK1* mutation

**Fig. 2.**
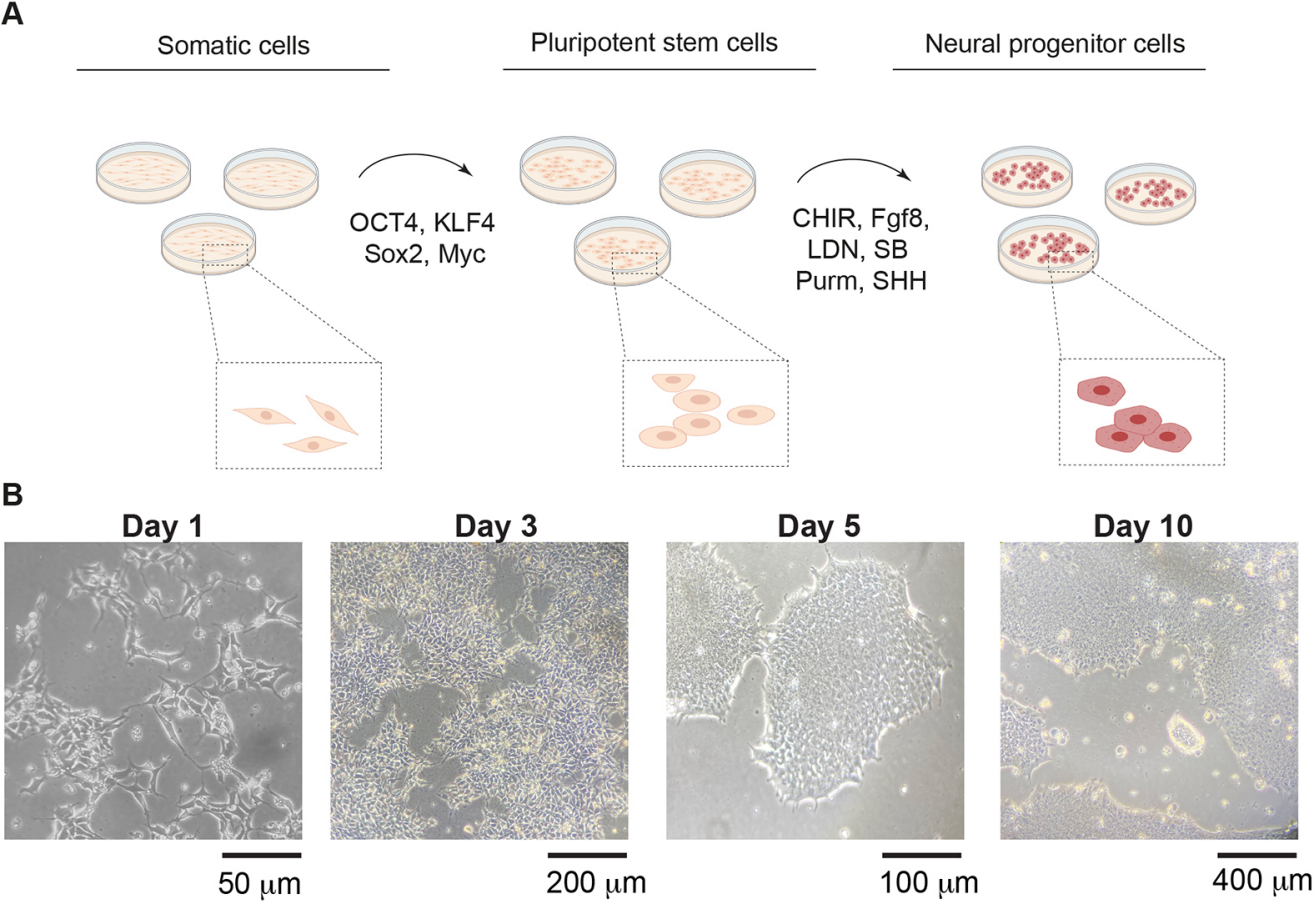
**Characterisation of iPSC-derived NPCs.** (A) Outline of the neural differentiation protocol used to obtain neural precursor cells (NPCs) from human somatic cells via cell reprogramming. Further details can be found in Materials and Methods. Illustration was created using BioRender. CHIR, CHIR99021; LDN, LDN193189; Purm, purmorphamine; SB, SB431542; SHH, SHH-C24II. (B) Representative phase-contrast images showing the overall morphology of cells across the differentiation timeline. Images are from three independent experiments.

**Fig. 3.**
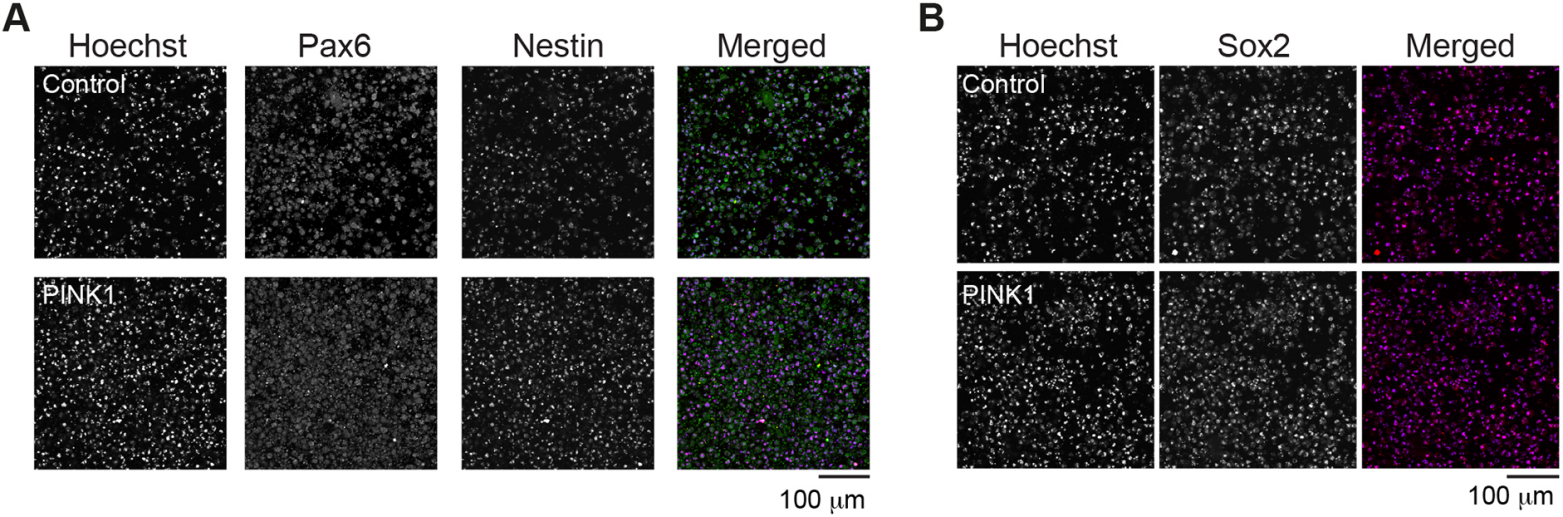
**Analysis of neural progenitor markers in *PINK1*-mutant NPCs.** (A,B) Immunofluorescence images showing the levels of Pax6 and nestin (A) or Sox2 (B) in control and *PINK1*-mutant NPCs (PINK1). Images are from three independent experiments. Control, CRISPR–Cas9-corrected N368I; PINK1, PINK1 I368N.

Next, we sought to determine whether the alterations in cysteine metabolism that were observed in *pink1*-mutant flies are also present in human cells with PINK1 mutations. We obtained fibroblasts from a female PD patient carrying a PINK1 I368N mutation (PINK1 cells) as well as isogenic fibroblasts in which this mutation was corrected to the PINK1 wild-type version by using CRISPR technology (control cells). These fibroblasts were first reprogrammed into iPSCs and then differentiated into NPCs following a previously published protocol (Kriks et al., 2011) (Fig. 2A). Both PINK1 and control cells were simultaneously differentiated into NPCs to compare their differentiation efficiency. Upon thawing and expansion, both lines demonstrated typical iPSC morphology, forming round colonies in a time-dependent manner, with little or no spontaneous differentiation detected around the colony borders. (Fig. 2B). Following 12 days of differentiation, we assessed the expression of neural progenitor markers nestin, transcription factor SOX-2 (Sox2) and paired box protein Pax-6 (Pax6), by immunofluorescence (Fig. 3A,B). We found similar levels of these markers in both control and *PINK1*-mutant NPCs (hereafter referred to as *PINK1* NPCs) and no significant difference of expression levels between groups.

### *PINK1* NPCs have a reduced mitochondrial membrane potential and ATP level

**Fig. 4.**
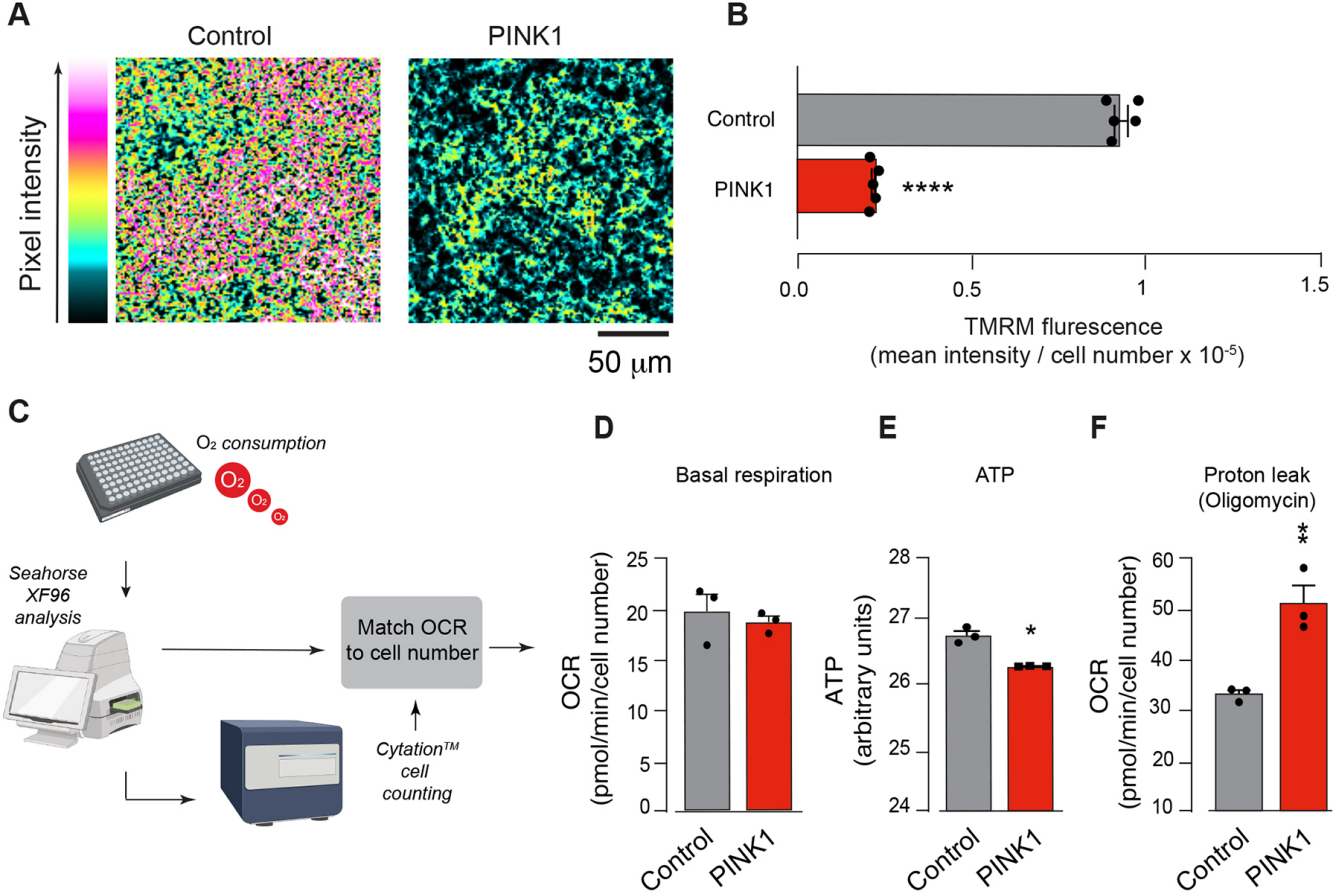
***PINK1*-mutant cells show mitochondrial deficits and increased respiration.** (A) Representative immunofluorescence images showing a loss in mitochondrial membrane potential (Δσm) in *PINK1-*mutant NPCs (PINK1). (B) Quantitative analysis of TMRM fluorescence for CRISPR–Cas9-corrected N368I (control) and PINK1 I368N (PINK1). Analysis was performed in iPSC-derived NPCs 12 days post differentiation. Intensity levels are visualised according to a five-tone heatmap (mean±s.e.m.; ****P<0.0001, unpaired Student's *t*-test, *n*=3). (C) Schematic of the workflow measuring the oxygen consumption rate (OCR) in NPCs. (D) Basal respiration levels of PINK1 NPCs are similar to those of control cells (mean±s.e.m.; unpaired Student's *t*-test, *n*=3). Each circle indicates the average OCR of five technical replicates per condition. Non-mitochondrial respiration was subtracted from each measurement. (E) *PINK1* NPCs show a significant decrease in cellular ATP. ATP levels were measured by mass spectrometry (mean±s.e.m.; *P<0.05, unpaired Student's *t*-test, *n*=3). (F) *PINK1* NPCs show an increased proton leak (mean±s.e.m.; **P<0.01, unpaired Student's *t*-test, *n*=3). Genotypes: control, CRISPR−Cas9-corrected N368I; PINK1, PINK1 I368N.

PINK1 plays important roles in mitochondrial homeostasis by ensuring the removal of defective mitochondria. The mitochondrial membrane potential (ΔΨm) is an indicator of mitochondrial function and health. To first assess the mitochondrial state in *PINK1* NPCs, we incubated cells with tetramethylrhodamine methylester perchlorate (TMRM) to label mitochondria and compared their basal ΔΨm to that of control cells. We found that *PINK1* NPCs had a significantly lower ΔΨm than control cells (Fig. 4A,B). Next, to determine whether the reduction in ΔΨm was linked to defects in respiration, we measured the mitochondrial oxygen consumption rate (OCR) using a Seahorse XFe96 Analyser (Fig. 4C). We first measured basal respiration in *PINK1* and control NPCs, and found no significant differences (Fig. 4D). However, we noticed a significant decrease in ATP levels (Fig. 4E).

Because mitochondrial respiration converts energy stored in macronutrients to both ATP and heat through natural proton leakage across the inner mitochondrial membrane, we next examined the rate of oxygen consumption independently of ATP synthesis. We incubated NPCs with the ATP synthase inhibitor oligomycin and measured their OCR (Dranka et al., 2011) and found that *PINK1* NPCs have an increased proton leak compared to that of control cells (Fig. 4F). We, therefore, believe that the basal oxygen consumption in *PINK1* NPCs is connected to increased proton leakage.

### *PINK1* NPCs show enhanced glycolysis

**Fig. 5.**
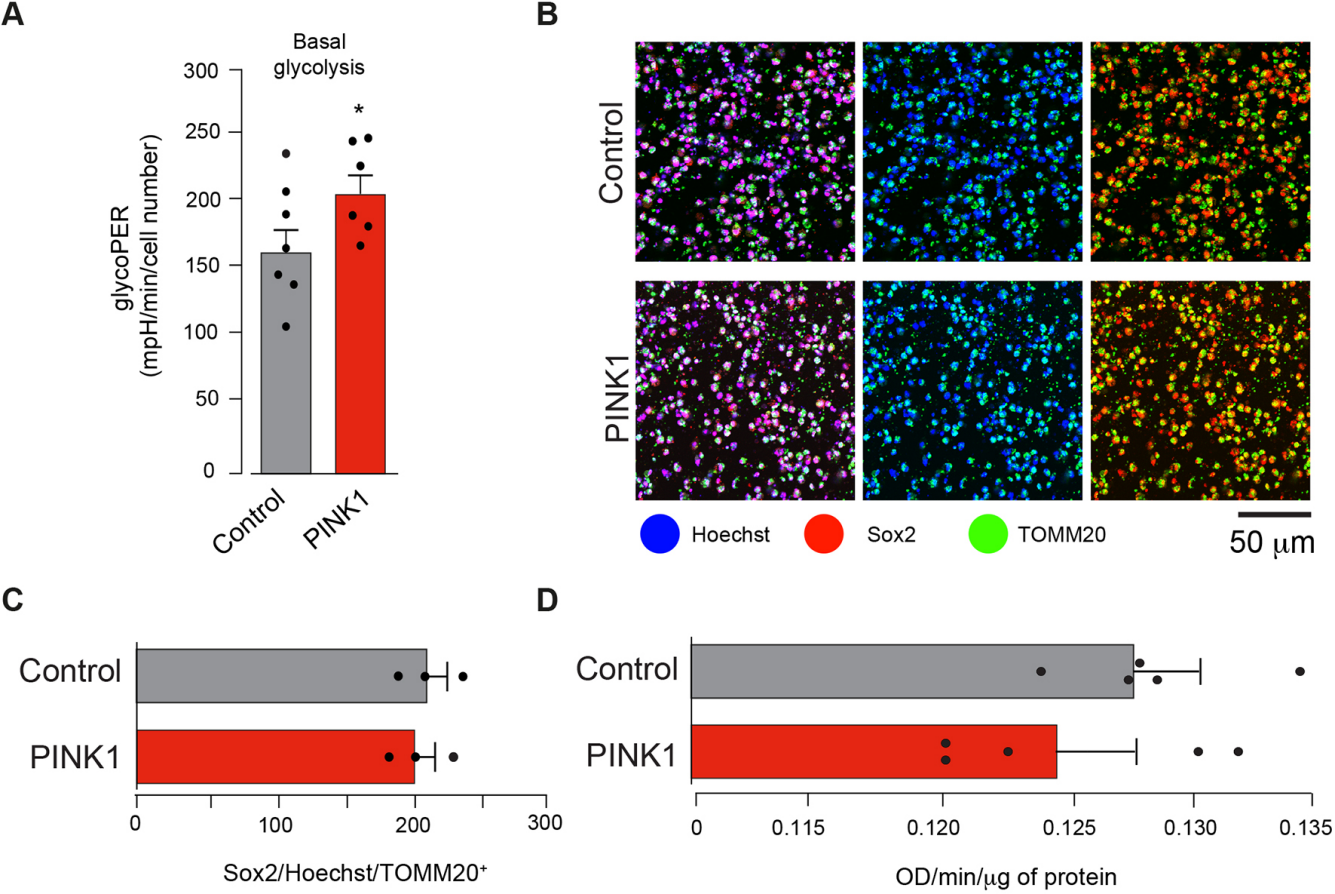
**Enhanced glycolysis in *PINK1*-mutant NPCs.** (A) Analysis of basal glycolysis in *PINK1-*mutant NPCs (PINK1) and control cells (mean±s.e.m.; *P<0.05, unpaired Student's *t*-test, *n*=6-7) as determined by Seahorse ECAR assay. Each circle indicates the average glycolytic proton efflux rate (glycoPER). (B-D) Assessment of mitochondrial mass in *PINK1*-mutant cells shows equivalent mass in control and *PINK1*-mutant NPCs. Mitochondrial mass was evaluated by either normalising levels of the mitochondrial marker TOMM20 to those of the transcription factor Sox2 (B,C) or by comparing the activity of the mitochondrial matrix enzyme citrate synthase in control and *PINK1*-mutant cells (D); unpaired Student's *t*-test, *n*=5. Representative immunofluorescence images (B) and quantitative analysis (C) in the indicated genotype. Analysis was performed in iPSC-derived NPCs 12 days post differentiation. Intensity levels of TOMM20 were normalised to those of Sox2 (mean±s.e.m., unpaired Student's *t*-test, *n*=3). Data are the result of three independent experiments. Genotypes: control, CRISPR−Cas9-corrected N368I; PINK1, PINK1 I368N.

Our findings show that, compared with control cells, *PINK1* NPCs exhibit mitochondrial impairment and a decreased cellular ATP production. Mitochondrial dysfunction in *Pink1*-knockout mice stimulates a compensatory metabolic response that increases glycolysis (Requejo-Aguilar et al., 2014). To determine if this compensatory increase in glycolytic activity is also recapitulated in *PINK1* NPCs, we next measured extracellular acidification, a readout of glycolytic activity (Mookerjee et al., 2016). Because production of CO_2_ due to mitochondrial activity can also contribute to extracellular acidification, we measured the glycolytic proton efflux rate (glycoPER) to determine the rate of protons extruded in the extracellular medium due to glycolysis (see Materials and Methods). Under basal conditions, this analysis showed that, glycolysis is elevated in *PINK1* NPCs (Fig. 5A).

PINK1 is a key effector of mitochondrial quality control as it promotes mitophagy of defective mitochondria in cells. To determine whether the loss of PINK1 in NPCs can lead to alterations in mitochondrial density, we next assessed mitochondrial mass by measuring the levels of the mitochondrial marker TOMM20. We did not detect alterations in TOMM20-positive cells (Fig. 5B,C). In addition, biochemical analysis of citrate synthase – an enzyme present in the mitochondrial matrix – did not detect significant differences between *PINK1* NPCs and control cells (Fig. 5D). Collectively, analysis of TOMM20 and citrate synthase levels indicated that differences in the mitochondrial mass do not explain the metabolic alterations in *PINK1* NPCs.

### *PINK1* I368N NPCs show alterations in cysteine metabolism

**Fig. 6.**
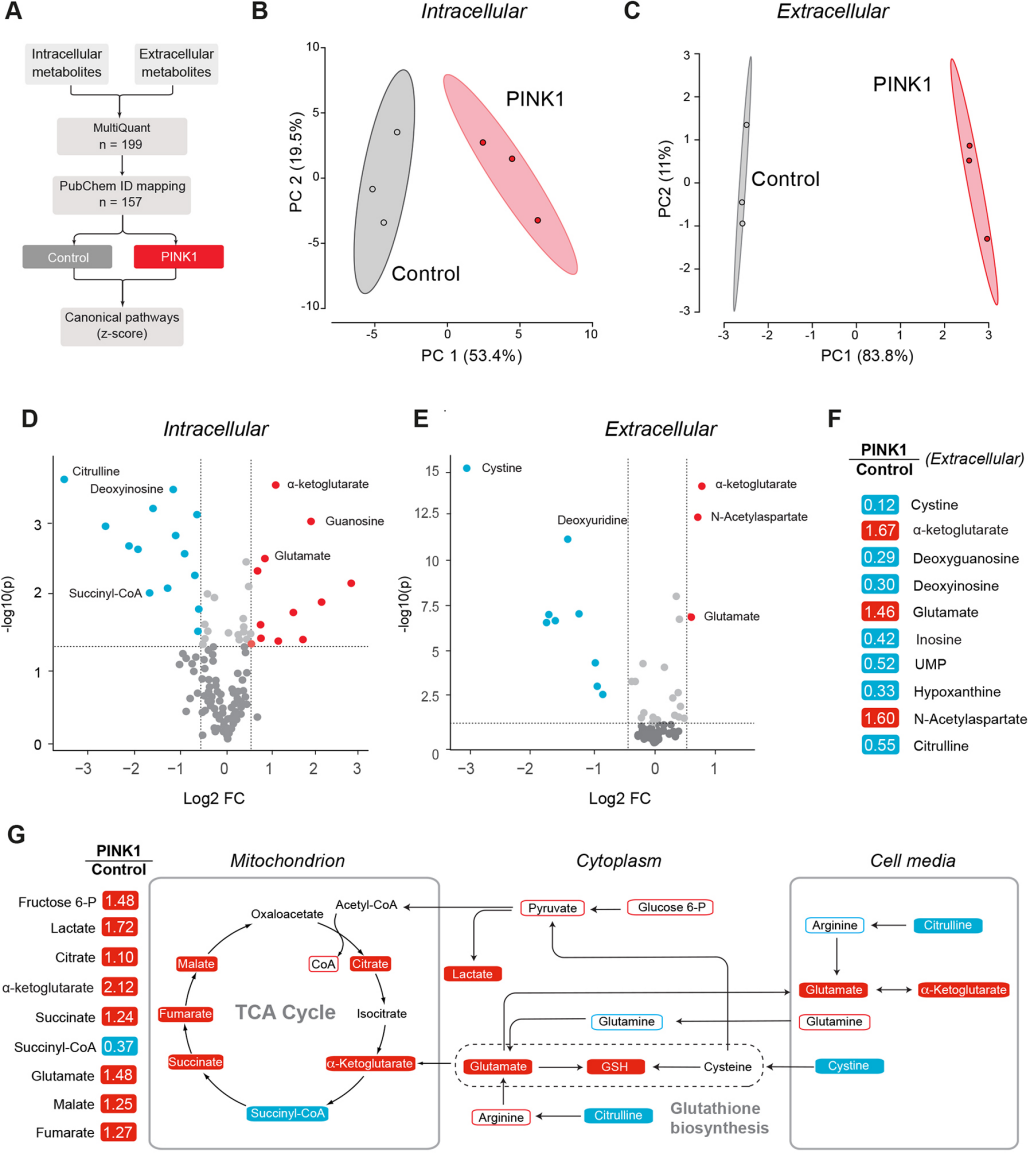
**Increased cysteine metabolism in *PINK1*-mutant NPCs.** (A) Workflow used for the identification of intracellular and intercellular metabolites by mass spectrometry. (B,C) Principal component analysis (PCA) of intracellular (B) and extracellular (C) global metabolic changes in *PINK1* NPCs (PINK1). (D,E) Significantly increased (red) or decreased (blue) intracellular (D) or extracellular (E) metabolites in *PINK1* NPCs. Significance was determined using Student's *t*-test; with *P*<0.05 considered significantly different and an absolute log2-fold-change >0.5. Dashed lines denote significance levels of metabolites above −(log10) *P*>1.3 and log2-fold changes of >0.5 or −0.5. (F) Altered metabolites in the extracellular space of *PINK1* NPCs. Red and blue correspond to significantly up- or downregulated metabolites, respectively (*P*≤0.05). Statistical significance was determined using Welch's two-sample Student's *t*-test (*n*=3). (G) Coordinated changes in the metabolite abundance in *PINK1* NPCs. Shown on the left are the relative levels of selected metabolites in the intracellular space of *PINK1* NPCs (detected with mass spectrometry and liquid chromatography with tandem mass spectrometry analysis). Data are the result of one experiment with three technical replicates. Genotypes: control, CRISPR−Cas9-corrected N368I; PINK1, PINK1 I368N.

Our analysis of *pink1*-mutant flies showed that mitochondrial dysfunction coincides with an increase in markers of cysteine metabolism and oxidative stress. To determine whether these metabolic alterations are also present in *PINK1* NPCs, we analysed their global metabolic profile, as illustrated in Fig. 6A. We conducted this analysis for both intracellular metabolites and for metabolites present in the culture medium (extracellular). In both cases, unsupervised clustering by using principal component analysis (PCA) showed a clear separation of the metabolic profile between the control and *PINK1* NPCs (Fig. 6B,C). After excluding internal standards, we detected a total of 157 individual metabolites in ∼95.4% of the samples that were included in our analysis. Of these, 46 metabolites were altered in the intracellular space (Fig. 6D, Table S1), and 23 were altered in the extracellular space (Fig. 6E, Table S2).

Intracellular metabolic profiling revealed an increase in several tricarboxylic acid (TCA) cycle metabolites in the *PINK1* cells (Fig. 6D-F), with the largest change observed for α-ketoglutarate (Fig. 6F). Glutamate can be converted to α-ketoglutarate through glutaminolysis, thereby compensating for losses in the TCA cycle in *pink1*-mutant flies (Tufi et al., 2014). We also detected an increase in both intracellular and extracellular glutamate in *PINK1* cells (Fig. 6D,E). Comparison of *PINK1* NPCs with controls further confirmed that purine metabolism intermediates, such as adenosine and guanosine (Table S1), were upregulated in *PINK1* NPCs, recapitulating previous changes detected in *pink1*-mutant flies (Tufi et al., 2014).

Although we were unable to detect intracellular cysteine by using our analytical pipeline (see Materials and Methods) (Liigand et al., 2017), our extracellular analysis revealed a significant decrease in cystine, i.e. in the oxidised cysteine dimer, in *PINK1* NPCs (log2-fold change: 0.12, *P*<0.0001; Fig. 6E-G). Because the availability of cysteine is the substrate-limiting step in glutathione synthesis (Ishii et al., 1987), we next investigated whether this could have an impact on glutathione levels in *PINK1* NPCs. We observed that *PINK1* NPCs contained significantly lower levels of reduced glutathione (GSH, log2-fold change: 1.47, *P*<0.05) and a mild but not significant increase in oxidised glutathione (GSSG, log2-fold change: 0.77, *P*=0.13, Table S1) compared to that of the control. GSH is one of the most important scavengers of ROS and is synthesised in a two-step reaction (Meister, 1988). Increased levels of GSH at the expense of GSSG may indicate increased oxidative stress in *PINK1* NPCs. We also detected a substantial change in the biochemicals associated with GSH biosynthesis (Fig. 6G), including glutamate and citrulline. The latter can be metabolised to arginine, a glutamate precursor, in an ATP-dependent reaction (Osowska et al., 2004). These findings indicate that compromised mitochondria in *PINK1* NPCs manipulate cysteine metabolic pathways to sustain higher levels of GSH biosynthesis. These data suggest that the cystine/cysteine cycle in *PINK1* NPCs fuels the bulk antioxidant function of cellular GSH. Taken together, the results indicate that both *pink1*-mutant flies and *PINK1* cells exhibited alterations in key networks associated with cysteine metabolism and glutathione biosynthesis. This supports a model in which increased glutathione biosynthesis is an early and predominant feature of Pink1 loss of function, pointing to a unifying perspective on early-stage PD pathology that could guide future therapeutic strategies.

## DISCUSSION

Studies of autosomal recessive mutations in *PINK1* have highlighted specific molecular defects of individual missense variants, of which the details have contributed to a better understanding of the pathway itself, its links to neurodegeneration and their overall relevance to PD. This has been particularly interesting not only for obtaining a complete understanding of early pathogenic events in PD but also for obtaining a rationalised future drug design.

Here, we used a computational pipeline to integrate diverse molecular datasets obtained from two PD models, *Drosophila pink1* mutants and patient-derived *PINK1* I368N NPCs. We showed that defects in PINK1 signalling cause global metabolic changes in iPSC-derived NPCs that broadly validate the comprehensive number of hits recovered in *pink1*-mutant flies, suggesting there is a substantial overlap between our integrated networks. Our data suggest that mitochondrial dysfunction upon loss of PINK1 function is accompanied by prominent changes in cysteine metabolism.

In this model of PD, a combined analysis of *pink1*-mutant flies uncovered metabolic and transcriptional modifications in cysteine metabolism associated with defects in mitochondrial respiration and increased ROS. These alterations paralleled the robust changes in biochemicals associated with cysteine metabolism and glutathione biosynthesis in *PINK1* NPCs, most likely as a result of oxidative stress. Previous studies have shown that the *PINK1* I368N mutation results in a complete loss of kinase activity (Ando et al., 2017) and a selective defect in dopamine metabolism (Novak et al., 2022). However, to date, no study has explored the global metabolic changes associated with this mutation. Our data support a role of this mitochondrial kinase in sustaining mitochondrial function. The data also support prior observations in other PD models, in which the loss of PINK1 had been linked to reduced Δσm, altered ATP synthesis and increased glycolysis (Cooper et al., 2012; Requejo-Aguilar et al., 2014; Villeneuve et al., 2016). Moreover, our data uncovered a role for cysteine metabolism in compensating for early and selective defects in mitochondrial respiration and ATP production.

Maintaining a healthy ratio of reduced to oxidised glutathione (GSH:GSSG) is essential for reducing oxidative damage and preventing cell death. Previous studies have reported increased oxidative stress and decreased GSH:GSSG ratios in the brain of PD patients (reviewed by Dias et al., 2013), but it remains unclear whether variations in cysteine levels can account for these deficits. By combining intracellular and extracellular metabolomics, we observed that robust cystine uptake coincides with increased GSH levels in *PINK1* NPCs, indicating that cells may utilise more cysteine to expand their GSH pool and mitigate oxidative stress. Although the literature is limited regarding the role of cysteine in PD, depletion of GSH is the earliest biochemical change described in the brain of PD patients (Perry et al., 1982). In one study, impaired mitochondrial function and GSH depletion are the earliest known indicators of substantia nigra degeneration in the brain of PD patients, and the magnitude of GSH depletion mimicked the severity of the disease (Jenner, 1998). Links between GSH deficiency and PD have stimulated research on approaches to maintain or restore GSH levels in these patients (Hauser et al., 2009). In particular, the use of N-acetyl cysteine (NAC), the N-acetyl derivative of cysteine, in PD has been proposed to increase cysteine availability and, thus, glutathione synthesis. Data from ongoing clinical trials indicate that administration of NAC increases binding of dopamine active transporter (DAT) in the caudate and putamen of PD patients, leading to a significant improvement in PD symptoms (Monti et al., 2019). NAC interferes with the recruitment of PINK1 to mitochondria under stress, indicating that this derivative of cysteine plays a role in controlling mitophagy (Gao et al., 2020). ROS can activate mitophagy and ROS scavengers, including NAC, and can suppress mitochondrial toxicity and mitophagy (Wei et al., 2015; Xiao et al., 2017; Xiong et al., 2019).

Combined multiomics approaches are limited because it is difficult to establish a causative role for the observed changes. Instead, the changes could represent a cellular homeostatic adaptive response to physiological damages. Using *Drosophila* genetics guided by network analysis and cells from a PD patient enabled us to validate specific metabolic signatures and to identify cysteine metabolism as a PD-associated metabolic signature. Despite these advantages, we must highlight a few caveats in our study.

First, when identifying novel metabolic signatures, mass spectrometry coupled with liquid chromatography (LC) is often used to obtain more-comprehensive phenotyping. The use of a negative ion mode for our metabolomics analysis prevents the direct detection of cysteine. However, measuring detectable levels of unpaired cysteine residues presents notable challenges, as this amino acid can undergo enzymatic or oxidative post-translational modification in response to changing redox conditions (Combs and DeNicola, 2019). This limitation may hamper definitive conclusions on cysteine metabolism in *PINK1* I368N NPCs, and future studies that explore cysteine–GSH interactions and glucose utilisation by using adequate isotope-labelled metabolic flux analysis are warranted.

To overcome the variability that arises from cell type heterogeneity linked to long-term differentiation protocols, we differentiated our iPSCs into NPCs, which also enabled us to detect early features of disease progression. However, recent evidence suggests that culture-medium-induced conditions can impact the metabolic profile of differentiating cells. Although both cell lines expressed neural progenitor markers, it cannot be excluded that the observed changes in energy metabolism are the result of highly manipulated culture-induced conditions. More research should address these shortcomings by systematically comparing the metabolic profile of PINK1 I368N NPCs at different time points during neuronal differentiation. Furthermore, we suggest that future work is needed to determine whether the metabolic phenotype identified in this study is also present in flies where the endogenous *pink1* gene is replaced with PINK1 I368N or in iPSC-derived NPCs with a homozygous deletion for *PINK1* (PINK1 knockout).

Our results show that the defects in mitophagy caused by *PINK*1 mutations reprogram cysteine metabolism to counteract the increases in oxidative stress triggered by the accumulation of defective mitochondria. Although the genetic models used in this study fail to fully recapitulate sporadic forms of PD, mitochondrial dysfunction is often observed in both genetic and sporadic PD (reviewed by Celardo et al., 2014). Therefore, it is conceivable that the mitochondrial defects reported in sporadic PD can also be associated with the metabolic phenotype identified in our study.

Finally, the methodology used in this study provides a framework for the analysis of other neurodegenerative diseases.

## METHODS

### Genetics and *Drosophila* strains

Fly stocks and crosses were maintained on standard cornmeal agar medium at 25°C. The strains used were *pink1^B9^, park^25^* and *daGAL4* (a kind gift from A. Whitworth, MRC, Centre for Developmental and Biomedical Genetics, University of Sheffield, Sheffield, UK). *w1118* was from Bloomington Stock Centre (Bloomington, IN, USA). All experiments on flies were performed using males.

### Generation of human p.I368N PINK1 iPSCs

Cells were derived from a homozygous carrier of the PINK1 c.T1103A (p.I368N) mutation, a mutation recently identified in Polish early-onset PD patients (Koziorowski et al., 2013). Because line-to-line variability causes a prominent challenge in identifying even subtle disease phenotypes in stem cell-derived PD models, control cells consisted of an isogenic CRISPR–Cas9-corrected cell line derived from the same p.I368N PINK1 donor (Subject ID NDS00227). Both cell types were shipped from Coriell Institute (Camden, NJ, USA) – now part of the NINDS Human Cell and Data Repository – where they had been authenticated and tested for contamination. Reprogramming and characterisation (pluripotency, stability of karyotype and differentiation potential) was performed by the Coriell Institute. The patient, a Caucasian 60-year-old female, had been reported to have exhibited early-onset and slowly progressive PD that started at a relatively young age (28 years), consistent with the typical clinical presentation of homozygous PINK1 loss-of-function. Some clinical features detected in this patient, including resting tremor, bradykinesia and laterality of parkinsonism, were indistinguishable from sporadic late-onset PD. Other clinical symptoms included mild autonomic failure (e.g. constipation) but neither dementia nor depression and anxiety were observed, which are occasionally recognised as a non-motor symptom of PINK1-associated PD.

### iPSC expansion and differentiation into NPCs

Skin cells from a homozygous carrier of the PINK1 I368N mutation were reprogrammed by the Coriell Institute into iPSCs using a combination of Oct3/4, Sox2, Klf4 and c-Myc. In our laboratory, gene-corrected and PINK1 p.I368N IPS cells were differentiated into floor plate neural progenitors following a previously published protocol. Briefly, the protocol is based on neural induction by dual-SMAD inhibition (Chambers et al., 2009) and the sequential activation of SHH and WNT signalling (Kriks et al., 2011). Upon thawing (day 0), iPSCs were plated in clear-bottom, Geltrex-coated 6-well plates and incubated with E8 medium (Life Technologies, cat. no. A1517001) supplemented with 10 μM ROCK inhibitor Y27632 (Tocris) for the first 24 h. To remove the Y27632, ∼90% of the medium was completely replaced the next day and replaced with fresh E8 medium. Daily changes of medium continued until ∼80% confluence was reached. Next, iPSC lines transferred into 24-well plates (NUNC, USA) that had been precoated with Geltrex (Gibco) were differentiated towards neural-specific progenies under 21% O_2_ and 5% CO_2_ at 37°C, employing a modified dual SMAD protocol (Shi et al., 2012). Neural induction was initiated by changing the E8 medium to neural-induction medium containing 50% Neuro basal medium (ThermoFisher Scientific, cat. no. 10888-022), and 50% DMEM/F12 (ThermoFisher Scientific, cat. no. 10565018) supplemented with B-27 (ThermoFisher Scientific, cat. no. 12587-010, 1:50) and N-2 (ThermoFisher Scientific, cat. no. 17502-048, 1:100). As previously described (Kriks et al., 2011), neural induction was then based on the timed exposure to SMAD inhibitors SB431542 (Selleck, cat. no. S1067, 10μM) and LDN193189 (Selleck, cat. no. S2618, 100nM), and to fibroblast growth factor 8 (FGF8, 100 ng/ml, R&D), CHIR99021 (3 μM, Stemgent), SHH-C24II (100 ng/ml, R&D) and purmorphamine (2 μM, Stemgent). Each reagent was added at different time points, as detailed by Kriks and colleagues (Kriks et al., 2011), i.e. between days 0 and 7 medium was supplemented with the dual SMAD inhibitors LDN193189 and SB431542. In addition, medium was supplemented with the SHH signalling inducers SHH-C24II, purmorphamine and FGF8 between days 1 and 6. Inhibition of Wnt signalling was induced by adding the GSK3β inhibitor CHIR99021 to the medium between days 3 and 12. All experiments were carried out at least 12 days from the start of the differentiation protocol for both cell lines, i.e. ∼30 days post iPSC thawing).

### Antibodies and dyes

Primary antibodies and dyes employed in this study were anti-nestin (Abcam, cat. no. ab22035, 1:1000), anti-Sox2 (ThermoFisher Scientific, cat. no. 149811-82, 1:200), anti-Pax6 (Biolegend, cat. no. PRB-278P, 1:100) and anti-TOMM20 (ThermoFisher Scientific, cat. no. PA5-52843, 1:100). Secondary antibodies were goat anti-mouse IgG H&L (Alexa Fluor 555) (Abcam, cat. no. ab150118, 1:500) and goat anti-rabbit IgY H&L (Alexa Fluor 488) (Abcam, cat. no. ab 150077, 1:500). Nuclei were visualised using Hoechst 33342 stain (ThermoFisher Scientific, cat. no. 62249, 1:10,000).

### Immunofluorescence and confocal microscopy of NPCs

NPCs were washed three times with PBS before fixation with 2% (v/v) paraformaldehyde (PFA) in PBS for 20 min. Before immunostaining, cells were blocked with 3% (w/v) bovine serum albumin (BSA) and permeabilised with 0.3% (v/v) Triton X-100 for 1 h at room temperature. Primary antibodies were diluted in 3% (w/v) BSA and incubated overnight at 4°C. Cells were analysed using a confocal microscope (LSM 510 Meta, Zeiss).

### Mitochondrial oxidative consumption rate

Mitochondrial oxygen consumption rates (OCRs) were recorded in IPSC-derived NPCs using the Seahorse XFe96 extracellular flux Analyser (Agilent). One hour before the start of the recordings, neural-induction medium was changed to Seahorse XF Base medium (cat. no. 102353-100, Agilent), supplemented with 5 mM glucose, 2 mM glutamine, 1 mM sodium pyruvate, 0.5 mM HEPES and 80 µg/ml carnitine pH 7.4. Cells were incubated at 37°C for 60 min to allow the medium temperature and pH to reach equilibrium before the assays were started. For the basic mitochondrial stress test, cells were seeded in Geltrex-coated Seahorse XFe96 plates at day 1 of neural induction. During the experiment, the OCR was measured before any addition of mitochondrial toxins. This is referred to as the basal state (with the base medium containing glucose, pyruvate and glutamine). Sequentially added to the wells were then 1.5 µM oligomycin (Santa Cruz Biotechnology) ‘minimal respiration’, 5 µM of the mitochondrial oxidative phosphorylation uncoupler FCCP (Santa Cruz Biotechnology) ‘maximal respiration’ and 1 µM antimycin A (Santa Cruz Biotechnology)/1 µM rotenone (Sigma Aldrich) ‘mitochondrial inhibition’. The last addition, i.e. of the mitochondrial respiratory chain (MRC) complex I blocker rotenone and MRC complex III blocker antimycin A caused complete inhibition of mitochondrial respiration, thereby allowing us to probe non-respiratory oxygen consumption. The mitochondrial OCR was measured every 2 min for a total of 65 min. The OCR was then used to estimate basal and maximal respiration, ATP synthesis and protein leakage as previously described (Underwood, et al., 2020). Following respiratory analysis, medium was removed and the cells were fixed with 4% (w/v) PFA containing Hoechst 33342 stain (1:10,000, ThermoFisher Scientific) for 15 min, before washing and imaging for automated cell counting. After completion of the Seahorse assay, normalisation was performed through fluorescence imaging of Hoechst 33342-stained nuclei to determine cell numbers in the XFe96-well plates. Blue Hoechst 33342 stain-positive cells were acquired and counted using a Cytation™ Cell Imaging Multi-Mode Reader. Similar to previous reports, the minimum OCR after addition of rotenone and antimycin was interpreted as the OCR resulting from non-mitochondrial respiration.

### Glycolytic rate assay

Unlike the Seahorse Mito Stress assay, the Seahorse XF Glycolytic Rate Assay utilises both extracellular acidification rate (ECAR) and OCR measurements to determine the glycolytic proton efflux rate (glycoPER) of the cells. Compared to the available ECAR measurements, this technique has notable advantages, i.e. that ECAR measurements are based on the assumption that the extrusion of lactate constitutes the principal source of extracellular acidification. However, the CO_2_ production resulting from mitochondrial activity can also contribute to extracellular acidification (Gohil et al., 2010). Mitochondrion-derived CO_2_ can partially hydrate in the extracellular medium, yielding H^+^+HCO_3_, resulting in additional acidification of the extracellular medium (Mookerjee et al., 2015). The Seahorse XF Glycolytic Rate Assay uses a CO_2_ contribution factor (CCF), an empirically derived value to allow the conversion of mitochondrial respiration, i.e. the mitochondrial OCR, into the CO_2_-dependent proton efflux rate (PER) to reduce the CO_2_ contribution to PER. To do this, the assay uses a standard contribution rate of CO_2_ to acidification that can be experimentally determined in multiple cell lines and primary cell cultures, including neural progenitors. The resulting value, the glycolytic proton efflux rate (glycoPER), is the rate of protons extruded in the extracellular medium that is specific to glycolysis.

First, cells were incubated in Seahorse XF Glycolytic Rate Assay Medium containing substrates, such as glucose (1 mM), glutamine (2 mM), and pyruvate (1 mM), as well as HEPES buffer and basal rates were recorded over three measurement periods. Second, a mixture of 1 µM rotenone and antimycin A (inhibitors of the mitochondrial electron transport chain) was injected to inhibit mitochondrial oxygen consumption and, therefore, CO_2_-derived protons. The second injection is 2-deoxy-D-glucose (50 mM), a glucose analogue that inhibits glycolysis by competitively binding glucose hexokinase, the first enzyme in the glycolytic pathway. ECAR, glycoPER, basal glycolysis and compensatory glycolysis were calculated as previously described (Selig, et al., 2019). Post-XF assay normalisation was performed using fluorescent imaging of Hoechst 33342-stained nuclei to determine cell numbers in the 96-well plates of the Seahorse XFe96 analyser. Blue Hoechst 33342 stain-positive objects were acquired and counted using the Cytation Cell Imaging Multi-Mode Reader. ECAR measurements were converted into PER, and mitochondrial OCR (baseline OCR – rotenone÷antimycin A) was used to determine the PER attributed to glycolysis (glycoPER) and mitochondrial (mitoPER) acidification using the Agilent Seahorse XF Glycolytic Rate Assay Report Generator and the Buffer (BF, 2.2) and CO2 contribution factors (CCF, 0.61) predetermined by Agilent.

### TMRM staining of mitochondria

To measure the mitochondrial membrane potential (Δσm) in the human NPCs, the cells were loaded with 50 nM tetramethylrhodamine methylester perchlorate (TMRM, T668 ThermoFisher Scientific) for 30 min at room temperature. IncuCyte S3 (Live-Cell Analysis System, Essen BioScience) was used for automated fluorescence microscopy analysis of TMRM uptake in mitochondria. In these experiments, TMRM was used in the redistribution mode to assess the Δσm and, therefore, a reduction in TMRM fluorescence represents mitochondrial depolarization.

### Measurement of citrate synthase activity

Activity of citrate synthase in IPSC-derived NPCs was determined using the Citrate Synthase Activity Assay Kit (Abcam, cat. no. ab119692). Data were measured as a change in absorbance per min per μg of protein after measuring the exact input protein per well. The measurements were obtained by kinetics reading at 412 nm for 10 min with shaking every 30 s using an EnVision^®^ 2105 multimode plate reader.

### Measurement of mitochondrial ROS in adult *Drosophila* brain

Brains of 3-day-old male flies were dissected in cold PBS and incubated with 5 μM MitoSOX Red mitochondrial superoxide indicator (M36008, Molecular Probes) for 30min as previously described (Garrido-Maraver et al., 2019). After incubation, brains were washed with PBS for 10 min, immediately imaged on a Zeiss LSM880 confocal microscope, and 89-μm-thick stacks were acquired. Maximal intensity projections of the MitoSOX signal in fly midbrain were quantified using ImageJ.

### Microarray analysis

Microarray analysis on 3-day-old male files was performed as previously described (Tufi et al., 2014). Briefly, RNA was prepared from 3-day-old male adult flies (24 samples in total, 12 replicates for each genotype). The RNA quality was confirmed using an Agilent 2100 Bioanalyzer. The target samples were prepared following the GeneChip One-Cycle Target Labelling protocol (Affymetrix). All samples were then hybridised to GeneChip *Drosophila* Genome 2.0 arrays (Affymetrix). The arrays were washed and stained using Affymetrix protocols on a Fluidics Station 400, scanned using a Gene Array Scanner 2500 and deposited at ArrayExpress under accession number E-MEXP-3645. Differential expression was analysed by the UK *Drosophila* Affymetrix Array Facility (University of Glasgow) with RankProducts and iterative Group Analysis using the automated FunAlyse pipeline.

### Metabolic profiling

For 3-day-old flies, global metabolic profiles were obtained using the Metabolon Platform (Metabolon Inc., NC, USA) as previously described (Tufi et al., 2014). Briefly, each sample consisted of eight biological replicates (100 flies per replicate). Sample preparation process was carried out using the automated MicroLab STAR^®^ system (Hamilton Robotics, Reno, NV, USA). For sample extraction, an 80% (v/v) methanol/water solution was used. Samples were then prepared for the appropriate analysis (either LC/MS or GC/MS). Compounds above the detection threshold were identified by comparison to library entries of purified standards or recurrent unknown entities present in Metabolon's proprietary LIMS system. Identification of known chemical entities was based on comparison to metabolomics library entries of purified standards.

To measure the global metabolic profiles in the human NPCs (three replicates per genotype), we first removed cell the neural-induction medium of each well and stored it at −80°C for further analysis. Cells were then quenched in a solution containing 400 μl of ice-cold (−20°C) acetonitrile, methanol and water (40:40:20) for 10 min. Next, the cell suspensions were transferred to 1.5 ml tubes and centrifuged at 21,000 ***g*** for 5 min at 4°C. Following centrifugation, the aqueous supernatant was collected and stored at −80°C. Chromatographic separation was performed as previously described (Michopoulos et al., 2014), and high-resolution metabolite mass detection was obtained on a Triple TOF 6600 mass spectrometer operated in negative ion mode (ESI-) and controlled through Analyst 1.8 software (AB Sciex, Warrington, UK). In-source instrument parameters were adjusted to Gas 1 60 (arbitrary unit), Gas 2 50 (arbitrary unit), curtain gas 35 (arbitrary unit), source temperature 500°C and ion spray voltage −4500 V. The raw spectrometric data were integrated with MultiQuan 2.0.2 (Applied Biosystems/MDS Sciex, Warrington, UK), and results exported to Excel for normalisation and univariate statistical analysis by Student's *t*-test. Peak areas from three biological replicates were averaged and ratios of the averages reported as fold changes between two cell lines. Metabolites with a coefficient of variation <30% in QC samples, *P*<0.05 and log2-fold changes of ≥0.5 or ≤−0.5 were considered significant. To ensure metabolite identity, the retention time of each analyte was compared to the retention time of the aqueous standard and spiked standard to the biological matrix of interest. If a metabolite was only detected in two of three samples, the zero value was excluded from all calculations.

Positive metabolite acquisition was performed on a TSQ Vantage controlled through Xcalibur 3.0.63 software (ThermoFisher Scientific, UK). In-source instrument parameters were adjusted to sheath gas 60 (arbitrary unit), auxiliary gas 20 (arbitrary unit), capillary temperature 350°C and ion spray voltage 3500 V. The binary gradient elution profile consisted of solvent A 0.05% heptafluorobutyric acid+0.05% acetic acid v/v in water with solvent B 100% MeOH. Metabolites were resolved on an HSS T13 1.8 μm 2.1×100 mm column, thermostated at 35°C under a flow rate of 0.4 ml/min with the following gradient elution program; 0 min 100%A, 0.5 min 100% A, 3 min 87% A, 4 min 87%A, 9 min 50% A, 9.1 min 0% A, 11.6 min 0%A, 11.7 min 100% A kept up to 16 min. Metabolite peak area data were extracted using Tracefindeer 5.1, and data exported to an Excel file for further analysis as described earlier.

### Network analysis

Fold changes obtained from Metabolon (for flies) and MultiQuan 2.0.2 software (for NPCs) were imported into QIAGEN Ingenuity^®^ Pathway Analysis (IPA^®^, QIAGEN Redwood City, US) to establish metabolic networks associated with differentially expressed metabolites. The canonical pathway analysis algorithm in IPA associates the probe sets with the canonical pathways in Ingenuity's Knowledge Base and returns the following statistical measures: (i) the ratio between the number of genes from the list that maps to the pathway and the total number of genes that map to the same pathway and (ii) a *P* value of Fisher's exact test. The activation *z* score was also used to infer the probable activation states of each pathway based on comparison with a model that assigns random regulation directions.

### Statistical analyses

Statistical analyses were performed using GraphPad Prism (www.graphpad.com). Data are presented as mean values, error bars indicate±standard error of the mean (±s.e.m.) or±standard deviation (±s.d.) The number of biological replicates per experimental variable (*n*) is indicated in either the respective figure or figure legend. For experiments conducted using the Seahorse XFe96 Analyser (Agilent), data were processed using Wave software (Agilent) using a CO_2_ contribution factor (CCF) of 0.61, as calculated for XFe96 by Agilent for <20 cell lines, including neuronal progenitors, and standardised to the cell number (per 1000 cells). Classification analysis, including PCA, and univariate plots (volcano plots) of metabolites were performed using the MetaboAnalyst 4.0 package in R (Chong et al., 2018).

When an insufficient number of cells was identified in one of the wells due to inaccurate plating or differential iPSC viability, the sample was considered an outlier and was removed from the analysis. Considered significant were *P<0.05, **P<0.01, ***P<0.001 and ****P<0; considered not significant was *P*≥0.05.

### Digital image processing

Fluorescence and transmission electron microscope images were acquired as uncompressed bitmapped digital data (TIFF format) and processed using Adobe Photoshop, employing established scientific imaging workflows (Sedgewick, 2008). Schematic workflows showing the iPSC differentiation protocol and the Seahorse apparatus were created using BioRender.

## Supplementary Material

10.1242/dmm.049727_sup1 Supplementary information
