# Supplementary material for: Increased cysteine metabolism in PINK1 models of Parkinson's disease
Source: Dis Model Mech. 2023 Jan 25;16(1):dmm049727. doi: 10.1242/dmm.049727 (PMC9903142; doi:10.1242/dmm.049727)
Supplement: Supplementary information [file dmm-16-049727-s1.pdf]

Table S1. Identification of significantly changed metabolites in the intracellular space of PINK1 I368N neural precursor cells. This table is related to Figure 6D.

Click here to download Table S1

Table S2. Identification of significantly changed metabolites in the extracellular space of PINK1 I368N neural precursor cells. This table is related to Figure 6E and F

Click here to download Table S2
